# Impact of Intramammary Treatment on Gene Expression Profiles in Bovine *Escherichia coli* Mastitis

**DOI:** 10.1371/journal.pone.0085579

**Published:** 2014-01-14

**Authors:** Anja Sipka, Suzanne Klaessig, Gerald E. Duhamel, Jantijn Swinkels, Pascal Rainard, Ynte Schukken

**Affiliations:** 1 Department of Population Medicine and Diagnostic Sciences, College of Veterinary Medicine, Cornell University, Ithaca, New York, United States of America; 2 Department of Biomedical Sciences, College of Veterinary Medicine, Cornell University, Ithaca, New York, United States of America; 3 GD Animal Health Center, Deventer, The Netherlands; 4 INRA, UMR1282, Infectiologie Animale et Santé Publique, Nouzilly, France; 5 Université François Rabelais de Tours, UMR1282 ISP, Tours, France; University of Queensland, Australia

## Abstract

Clinical mastitis caused by *E. coli* accounts for significant production losses and animal welfare concerns on dairy farms worldwide. The benefits of therapeutic intervention in mild to moderate cases are incompletely understood. We investigated the effect of intramammary treatment with cefapirin alone or in combination with prednisolone on gene expression profiles in experimentally-induced *E. coli* mastitis in six mid-lactating Holstein Friesian cows. Cows were challenged with *E. coli* in 3 quarters and received 4 doses of 300 mg cefapirin in one quarter and 4 doses of 300 mg cefapirin together with 20 mg prednisolone in another quarter. At 24 h (n = 3) or 48 h (n = 3) post-challenge, tissue samples from control and treated quarters were collected for microarray analysis. Gene expression analysis of challenged, un-treated quarters revealed an up-regulation of transcripts associated with immune response functions compared to un-challenged quarters. Both treatments resulted in down-regulation of these transcripts compared to challenged, un-treated quarters most prominently for genes representing Chemokine and TLR-signaling pathways. Gene expression of Lipopolysaccharide Binding Protein (LBP), CCL2 and CXCL2 were only significantly down-regulated in cefapirin-prednisolone-treated quarters compared to un-treated controls. Down-regulation of chemokines was further confirmed on the basis of protein levels in milk whey for CXCL1, CXCL2 and CXCL8 in both treatments with a greater decrease in cefapirin-prednisolone-treated quarters. The data reveal a significant effect of treatment on cell recruitment with a more pronounced effect in cefapirin-prednisolone treated quarters. Provided a rapid bacteriological clearance, combination therapy may prevent neutrophil-induced tissue damage and promote recovery of the gland.

## Introduction

Clinical mastitis is a highly prevalent disease in dairy cows and causes substantial economic loss for the dairy industry worldwide [1]. Infections with *E. coli* are assumed to be the most frequent cause of clinical mastitis on dairy farms with low somatic cell counts (SCC) [2], [3]. Although pathogen factors have an impact on infection dynamics [4], the amplitude of the inflammatory response is mainly dependent on individual cow factors [5], [6]. The efficacy of antibiotic and/or anti-inflammatory treatment in *E. coli* mastitis is still a topic of scientific debate. Treatment is generally recommended in severe cases and in the puerperal period [7]. Studies on treatment value in mild to moderate clinical cases however show conflicting results. No beneficial effect was observed for systemic treatment with trimethroprim-sulfadiazin or intramammary treatment with colistin in an experimental *E. coli* mastitis model [8]. Similar results were reported for systemic treatment with enrofloxaxin in combination with ketoprofen or ketoprofen alone in naturally occurring *E. coli* mastitis [9]. Others however, reported beneficial health effects when cephalosporin or fluoroquinolone antibiotics are used. An overall positive impact on clinical score, bacteriological cure and milk production after intramammary treatment was observed [3], [10], [11]. Despite the results in favor of treatment in mild to moderate cases, it is not generally recommended, mostly because of concerns for food safety and emergence of *E. coli* with antibiotic resistance [7], [12]. Less work has been done on the impact of glucocorticoids in combination with antibiotics in mastitis treatment. Systemic treatment with dexamethasone in endotoxin-induced mastitis reduced neutropenia, milk loss and the development of fever [13], [14]. Another study however indicated that systemic administration of isoflupredone did not have a beneficial effect on recovery in endotoxin-induced mastitis [15]. Due to the potential long term immune suppressive effects of glucocorticoids, the use of non steroidal anti-inflammatory drugs is usually recommended [12], [16].

The host immune response in intramammary *E. coli* infection has been studied intensively in recent years. Microarray based studies show a general up-regulation of genes associated with immune response mechanisms and down-regulation of genes related to fat metabolism in tissue of experimentally inoculated quarters [17], [18]. In both studies up-regulated genes show an enrichment of NF-κB regulated pathways, most prominently chemokine and cytokine signaling associated pathways within the first 24 h of the inflammatory response. This initial cytokine and chemokine response was shown to be a decisive mechanism during an *E. coli* mastitis and is known to play a critical role in the inflammatory process [19]. In an experimental mastitis model, it was shown that the strong up-regulation of TLR2 induced by *E. coli* challenge originated from the mammary gland epithelial cells [20]. In vitro challenge studies of cultured primary bovine mammary epithelial cells revealed a gene expression pattern in response to heat inactivated *E. coli* with the most prominent activation in genes encoding chemokines and cytokines [21]. To our knowledge, the outcome of intramammary therapy on the host chemokine and cytokine response patterns in udder tissue has not been investigated. In a previous study, we showed clinical and somatic cell count effects of intramammary treatment with cefapirin alone or in combination with prednisolone on the course of experimental *E. coli* mastitis [22]. Cefapirin-treated quarters showed a rapid bacteriological clearance and both treatments reduced local inflammation with the most prominent reduction in the influx of milk somatic cells and tissue polymorphonuclear neutrophil (PMN) infiltrate with the combination therapy. It remains unclear however which immune mediators are contributing to the treatment effects and were responsible for the treatment differences. The objective of the present study was to investigate the impact of intramammary treatment with cefapirin and prednisolone on immune response dynamics using microarray data to create gene expression profiles for challenged, treated and untreated quarters. We focused on the first 48 h post challenge, which covered the duration of the treatment protocol and enabled us to investigate potential treatment effects on the initial inflammatory response. A particular aim was to compare the expression profiles of antibiotic therapy alone versus a combination of antibiotics and glucocorticoids. To our knowledge this is the first assessment of udder tissue gene expression profiles as an approach to evaluate the efficacy of intramammary therapeutic interventions.

## Materials and Methods

### Ethics Statement

This study was approved by the Cornell Institutional Animal Care and Use Committee (project number: 2010-0078). All procedures involving animals were carried out in accordance with U.S. legislation on animal welfare. Cows were euthanized using a penetrating captive bolt followed by immediate exsanguination.

### Experimental Mastitis Model

Animal selection, challenge and sampling procedures were described in more detail in a previous report [22]. Briefly, six mid lactating Holstein Friesian cows were challenged with 100 colony forming units (cfu) of a fully characterized, cefapirin-susceptible *E. coli* mastitis strain (ECC-Z) [23] via the teat canal in 3 quarters. Treatment was performed 4 h after bacterial challenge. One of the rear quarters was treated with the combination of 300 mg cefapirin and 20 mg prednisolone (CFPD), the other rear quarter was treated with 300 mg cefapirin only (CF) (both MSD Animal Health, Boxmeer, the Netherlands). One quarter was challenged without treatment (CNT) and one quarter remained as un-challenged control (NC). Blood and milk samples were collected at challenge and every 6 h post challenge. Treatment was repeated at the subsequent milkings (12, 24 and 36 h post challenge). The cows were sacrificed for tissue collection at 24 (n = 3) and 48 h (n = 3) after infection. In total there were two treatments for cows being euthanized at 24 h post challenge, and four treatments for cows being euthanized at 48 h post challenge. Tissues samples were taken immediately after euthanasia. Samples for RNA preparation were obtained from the mid parenchyma of each quarter and immediately snap frozen in liquid nitrogen.

### Expression Profiling using Microarrays

Mammary gland tissue was snap frozen in liquid nitrogen and stored at −80°C until further assayed. Tissue was disrupted in lysis buffer using eppendorf pestles and RNA was extracted using the RNeasy kit (QIAGen, Gaithersburg, MD) as to the manufacturer’s instructions. Purity and integrity of the RNA was assessed using microfluidics analysis (2100 Bioanalyzer, Agilent). Only samples with an RNA integrity number >8.0 were used for further analysis, resulting in microarray analysis of tissues samples from 20 quarters of 5 cows. One cow was excluded from microarray analysis due to low integrity of collected RNA. Total RNA was amplified and biotin labeled (aRNA) with the MessageAmp™ Premier RNA Amplification Kit (Ambion®, Life Technologies, Carlsbad, CA). The obtained aRNA (15 µg per sample) was fragmented at 94°C for 35 min in a reaction volume of 30 µl containing 6 µl 5x array fragmentation buffer and nuclease free water following the manufacturer’s instructions. On completion of the fragmentation reaction, the hybridization cocktail was made according to the 49 Format with 5 µl of control oligonucleotide B2, 15 µl of 20X Eukaryotic Hybridization Controls, 3 µl of Herring Sperm DNA, 3 µl of BSA, 150 µl of 2x Hybridization Buffer, 30 µl of DMSO, and nuclease free water to a volume of 270 µl. This hybridization cocktail was then was added to the 30 µl of fragmentation mixture and a volume of 200 µl was hybridized to a GeneChip® Bovine Genome Array for 16 h at 45°C in a rotating Affymetrix hybridization oven. After hybridization the array was washed and stained using the GeneChip® Hybridization Wash and Stain Kit (all kits and Arrays were from Affymetrix, Santa Clara, CA). Slides were scanned immediately after washing on the Affymetrix GeneChip® Scanner 7G Plus, according to the manufacturer’s protocol. The Bovine Genome Array contains 24128 probe sets which represent 15264 UniGenes (annotation from May 2006) to measure the global transcripts. The Bovine Genome Array annotation available from NetAffx™ Analysis Center (Bovine.na29.annot.csv) was used. The microarray data has been deposited in the Gene Expression Omnibus database (accession number: GSE50685).

### Analysis of Microarray Data

The raw data was analyzed using Bioconductor software [24] in RStudio [25]. Normalization of expression values was performed using the Robust Multiarray Average (RMA) algorithm as implemented in the Affy package. Differential expression of each gene was assessed using empirical Bayes models which were implemented in the R package Limma [26]. Briefly, the model allowed for testing differences between treatment groups using a moderated Student’s t-test statistic to identify significantly differently expressed genes. This test moderates residuals of the standard deviations across the probe sets to obtain more stable interferences for each transcript. Probes were considered to be differentially expressed (DE) if the false discovery rate adjusted P-value was below 0.05 and had a minimum fold change of 2.

Similarity between samples was determined with the Gene Distance Matrix function in Multi-experiment Viewer (MeV version 4.8.01) [27] using Pearson correlation as a distance metric. Annotation and pathway analysis was performed using the online software tool DAVID (Database for Annotation, Visualization and Integrated Discovery) [28]. Overrepresentation of gene sets defined by the group of biological process (BP) in the Gene Ontology (GO) data base or the Kyoto Encyclopedia of Genes and Genomes (KEGG) pathway analysis were tested using Fisher’s exact test. Only significant DE genes which were annotated with an Entrez gene ID were included.

### Real Time PCR Analysis

Quantitative real time PCR (qPCR) was performed with the same RNA samples as used for the microarray hybridization. Synthesis of cDNA was performed with an amount of 1 µg total RNA using SuperScript II Reverse Transcriptase and oligo (dT) nucleotide primers (both Invitrogen, Life Technologies, Carlsbad, CA) according to the manufacturer’s instructions. Thermocycling was performed in a StepOnePlus Real Time PCR system with SYBR Green Master Mix (both Applied Biosystems, San Francisco, CA) using the following conditions: 95°C for 10 min, followed by 40 cycles of 95°C for 15 sec and 60°C for 60 sec with fluorescence detection during the annealing/extension step. The quality of amplification was verified by melt curve analysis. Primer sequences, references, accession numbers and concentrations used for amplification reactions are indicated in table 1.

**Table 1:** Primer sequences for quantitative RT-PCR.

Gene	Forward (for) and reverse (rev) primer sequences (5′→3′) and concentrations (nM)	bp^a^	NCBI Accession Nr.^b^
**CCL2**	for TCGCTGCAACATGAAGGTCT (300)	119	NM_174006.2
	rev TATAGCAGCAGGCGACTTGG (300)		
**CXCL2**	for GCCACTCTCAAGACTGGTCA (300)	152	NM_174299.3
	rev GGGCAGGGTCTACTTCTGGA (300)		
**LBP**	for GGTTCCGAAGGGTCTTGGAG (300)	166	NM_001038674.2
	rev AGCATTTGGGCTGTTGCTTG (300)		
**CXCL8**	for CCTCTTGTTCAATATGACTTCCA (300)	189	NM_173925.2
	rev GGCCCACTCTCAATAACTCTC (50)		
**IL-12 A p35**	for TGTCAGCAACACGCTACAGA (300)	124	NM_174355.1
	rev CAGTGGTAAACAGGCCTCCA (300)		
**UXT**	for CAGCTGGCCAAATACCTTCAA (300)	125	NM_001037471
	rev GTGTCTGGGACCACTGTGTCAA (300)		
**MRLP39**	for GCTGTGTGATAGAGAGGGCA (300)	96	NM_001080730.2
	rev CGTCATAGCAGAAGGCTCCA (50)		

^a^ bp, length of amplicons in base pairs.

^b^ NCBI National Center for Biotechnology Information.

### Analysis of qPCR Data

Two genes with known stable expression in udder tissue which were also not present on the list of differentially expressed genes from the microarray experiment, Ubiquitously expressed transcript (*UXT*) and Mitochondrial Ribosomal Protein L39 (*MRLP39*), were used as reference genes [29]. The cycle number required to achieve a definite SYBR Green fluorescence signal was calculated by the StepOne Software v2.3 (Applied Biosystems, San Francisco, CA) as cycle threshold (C_t_.) and is inversely correlated to the amount of target gene in the sample. The C_t_ of the reference genes showed no significant difference between all samples and a reference index was calculated to normalize the C_t_ of the target genes. The relative quantity (RQ) of the normalized target gene in samples of infected and treated quarters compared to the uninfected and untreated control quarters was calculated by the ΔΔC_t_ method in StepOne Software v2.3. The calculation was corrected by the efficiency of the primer pairs for each gene, which was determined beforehand and found to be between 96% an 114%. Statistical differences between RQ values from CNT and CF or CFPD quarters were analyzed with the non parametric NPAR1WAY procedure in SAS software Version 9.2 (SAS Institute Inc., Cary, NC).

### Sandwich Enzyme-linked Immunosorbent Assays (ELISAs) to Assess the Concentrations of Bovine Chemokines in Milk Whey

Milk samples were centrifuged at 20,000×g and 4°C for 30 min. The fat layer was removed and milk whey was collected and stored in aliquots at −20°C until ELISA analyses. Concentrations of the chemokines CXCL1, CXCL2 and CXCL8 were measured in milk by ELISA performed as previously described [30], [31]. Lower limits of detection in milk were 0.2, 0.1 and 0.01 ng/ml for CXCL1, CXCL2, and CXCL8, respectively.

## Results

### Challenge Induced Clinical Mastitis and Treatment Induced Rapid Bacteriological Cure

All cows developed clinical mastitis in the challenged untreated quarters. Body temperature was moderately elevated at 18 h post challenge (39.2±0.3°C, data not shown). Control quarters that were not challenged, did not develop mastitis and did not show presence of the challenge strain at any time point during the experiment. The challenge *E. coli* strain was isolated from milk from all challenged not treated quarters after challenge and at the time of tissue collection. Recovered isolates were confirmed to be the challenge strain by PCR-based fingerprinting [4]. At 24 h post challenge growth of the challenge strain could still be detected in the CF quarter of one cow. The other cows did not show bacterial growth in milk in any of the treated quarters. The clinical and bacteriological results have been described in more detail in Sipka et al. 2013 [22].

### Challenge Resulted in Up-regulation of Immune Response Associated Genes

Tissue samples from all 4 quarters of 2 cows at 24 h and 3 cows at 48 h post challenge were subjected to microarray analysis. Comparisons between CNT and NC quarters were made to observe the host response to *E. coli* ECC-Z at the respective time points. At 24 h post challenge, 231 DE genes (P<0.05) were identified of which 164 were up-regulated and 67 were down-regulated. At 48 h post challenge, a total of 290 DE genes (P<0.05) were identified of which 239 were up-regulated and 51 down-regulated (Tables S1, S2). Table 2 shows a representative selection of significantly enriched GO terms among up- and down-regulated DE genes in challenged not treated quarters at 24 and 48 h post challenge (P<0.05). Among up-regulated genes, the most enriched terms were associated with immune response functions, response to bacterium and cell activation (Tab. 2). Among down-regulated genes enriched terms included a lower count of genes and were mainly involved in fatty acid metabolic processes and lipid biosynthesis (Tab. 2).

**Table 2:** Significantly enriched Gene Ontology terms in up-regulated DE gene list (positive FI) and down-regulated DE gene list (negative FI) in challenged not treated quarters compared to not challenged quarters.

	24 h post challenge			48 h post challenge		
FI	GO Term	Count	P-Value^a^	GO Term	Count	P-Value^a^
**positive**	inflammatory response	22	6.14E-18	immune response	19	9.03E-09
	response to wounding	24	3.12E-15	inflammatory response	12	3.70E-08
	immune response	26	4.09E-12	response to wounding	13	1.05E-06
	response to bacterium	13	1.22E-08	chemotaxis	8	2.80E-06
	positive regulation of immune response	11	3.38E-07	response to bacterium	8	8.18E-05
	leukocyte activation	12	9.53E-07	locomotory behavior	8	1.83E-04
	positive regulation of immune system process	13	1.14E-06	positive regulation of response to stimulus	8	4.11E-04
	immune effector process	10	1.46E-06	leukocyte activation during immune response	4	9.99E-04
	cell activation	12	4.47E-06	proteolysis	17	0.002469
	lymphocyte mediated immunity	7	3.62E-05	regulation of phagocytosis	3	0.004823
**negative**	fatty acid metabolic process	5	0.0013	positive regulation of cholesterol storage	2	0.0075
	acylglycerol metabolic process	3	0.0049	fatty acid biosynthetic process	3	0.0082
	oxidation reduction	8	0.0049	lipid biosynthetic process	4	0.0082
	lipid biosynthetic process	5	0.0053	positive regulation of foam cell differentiation	2	0.0100
	glycerol ether metabolic process	3	0.0057	positive regulation of lipid storage	2	0.0100
	neutral lipid metabolic process	3	0.0057	regulation of cholesterol storage	2	0.0149
	organic ether metabolic process	3	0.0061	regulation of foam cell differentiation	2	0.0223
	monovalent inorganic cation transport	5	0.0109	organic acid biosynthetic process	3	0.0237
	cation transport	6	0.0161	carboxylic acid biosynthetic process	3	0.0237
	phosphate transport	2	0.0209	regulation of lipid storage	2	0.0272

^a^ significance of gene term enrichment examined by modified Fisher’s exact test.

### Challenged and Treated Quarters Showed High Similarity to Unchallenged Quarters at 24 and 48 h Post Challenge

**Figure 1:**
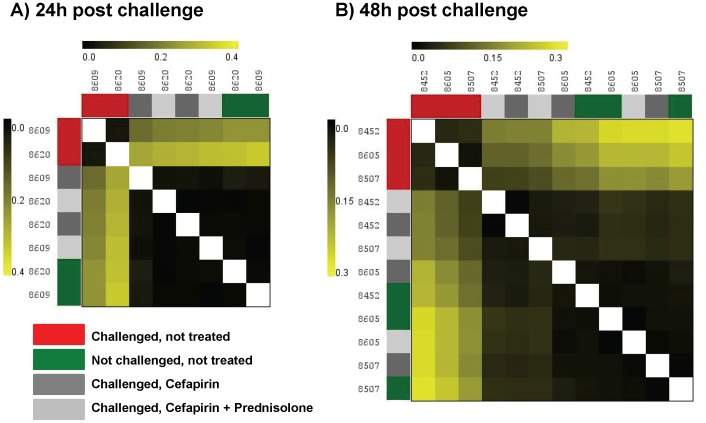
Hierarchical clustering of expression profiles. Mammary gland tissue was subjected to microarray analysis at 24(n = 2) and 48 h (n = 3) post challenge with 100 cfu *E. coli*. Per individual cow one quarter served as un-challenged control (green), one quarter was challenged but not treated (red) and two challenged quarters were treated at 4, 12, 24 and 36 h post challenge with 300 mg cefapirin (dark gray) or 300 mg cefapirin plus 20 mg prednisolone (light gray). Gene expression profiles of individual samples were compared and the degree of similarity of the expression patterns is shown as a heatmap of the distance matrix (metric: Pearson correlation). The darker the color (smaller distance) the more similar are the expression pattern between any two samples.

No DE genes could be identified between treated quarters and NC quarters at both time points. Hierarchical clustering of gene expression profiles of all samples showed a high similarity between NC and treated quarters while CNT quarters cluster together and show the least similarity to the rest of the samples (Fig. 1 A, B). Furthermore samples of individual cows showed a stronger overlap in gene expression at 48 h post challenge (Fig. 1 B). Compared to CNT quarters, a total of 171 annotated DE genes (P<0.05) could be identified in CF quarters at 24 h post challenge, among which 41 were up-regulated and 130 down-regulated (Table S3). At 48 h post challenge a total of 193 annotated DE genes was identified, with 23 up-regulated and 170 down-regulated annotated genes (Table S4). In CFPD quarters a total of 214 annotated DE genes could be identified at 24 h post challenge and 146 annotated DE genes at 48 h post challenge compared to CNT quarters with 161 down-regulated genes at 24 h post challenge and 133 down-regulated genes at 48 h post challenge (Tables S5, S6).

### Enriched Pathways in Down-regulated DE Genes of Treated Quarters are Associated with Cytokine Signaling and Cell Migration

**Figure 2:**
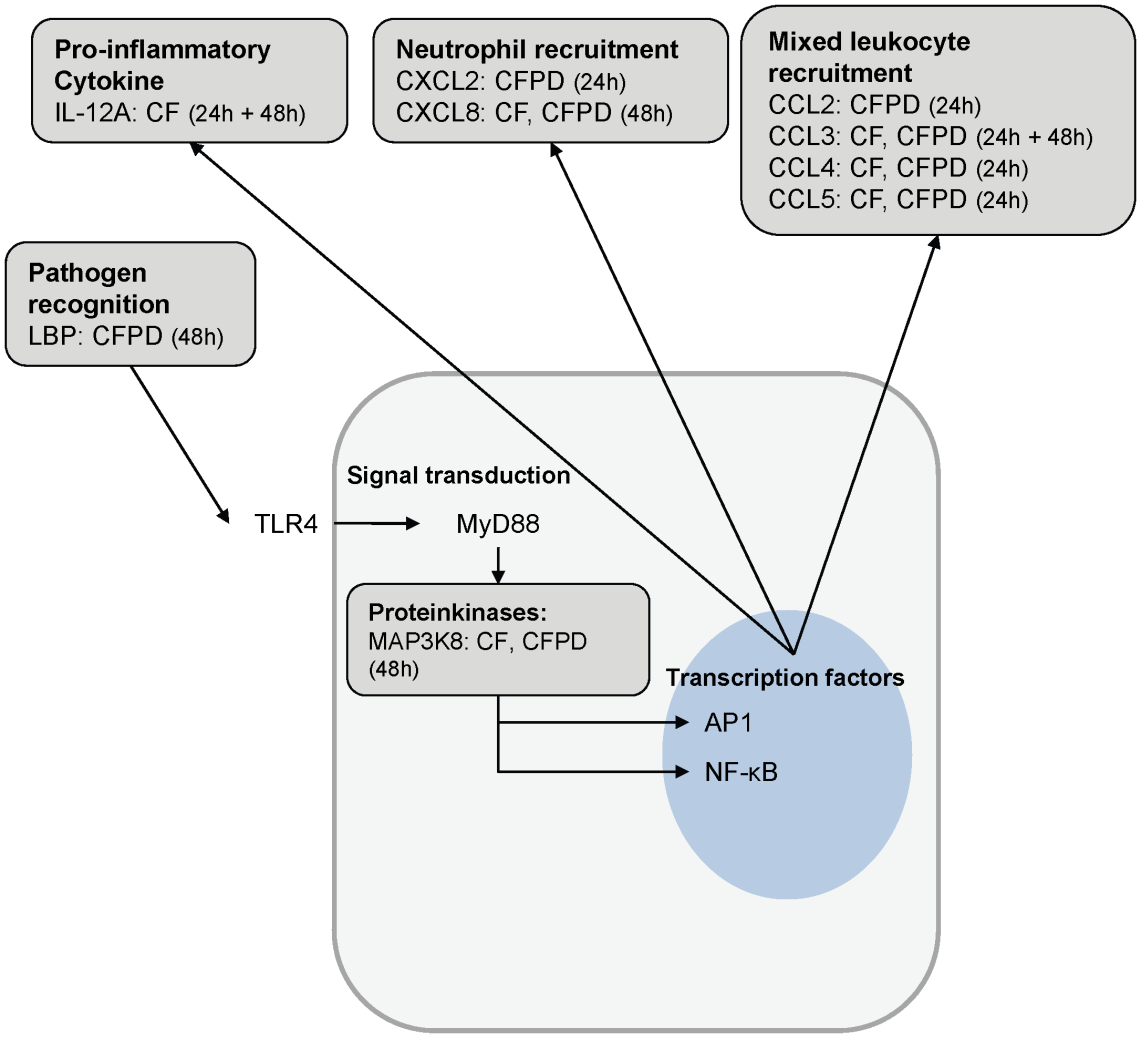
Influence of treatment on host immune response. Graphical summary of treatment mediated effects on selected genes representing the TLR- and Chemokine-signaling pathway. Genes listed are significantly down-regulated compared to challenged not treated quarters in quarters with the indicated treatment type at the indicated sampling time (C: cefapirin only, C+P: cefapirin plus prednisolone). Pathway organization adapted from KEGG. TLR4: Toll-like receptor 4 MyD88: Myeloid differentiation primary response 88 AP1: Activating protein 1 NF-κB: Nuclear Factor κB.

Down-regulated DE genes of treated quarters were subjected to pathway analysis through KEGG. The KEGG identifiers cytokine-cytokine receptor interaction (KEGG4060) and chemokine signaling pathways (KEGG4062) were found to be significantly enriched for both treatments (CF quarters: P<0.0001, P = 0.0305, CFPD quarters: P<0.0001, P = 0.0080) at 24 and 48 h post challenge. At 48 h post challenge the KEGG identifier Toll-like receptor (TLR) signaling (KEGG4620) was significantly enriched for both groups as well (CF: P = 0.0025, CFPD treated quarters: P<0.0001). Table 3 shows negative log 2 fold induction (FI) and P-values of DE genes belonging to the enriched pathways for both, treatments and time points, as well as their positive log 2 FI in CNT quarters. Relevant chemokine ligands belonged to the C-C and the C-X-C motif group. At 24 h post challenge *CCL3, 4* and *5* were significantly down-regulated in both treatment groups, while at 48 h post challenge only *CCL3* remained down-regulated in treated quarters. Among C-X-C motif ligands *CXCL8* was the top up-regulated gene in challenged not treated quarters at 24 h post challenge (FI 5.3) with no significant difference in regulation in treated quarters relative to the untreated challenged quarters. At 48 h post challenge CF and CFPD quarters showed a significant down-regulation of *CXCL8* transcripts relative to CNT quarters. Gene expression of *CXCL2* showed a different pattern. At 48 h post challenge *CXCL2* was the top up-regulated DE gene in CNT with no significant difference in regulation in treated quarters relative to CNT. While at 24 h post challenge *CXCL2* is moderately up-regulated in challenged, CNT quarters and significantly down-regulated in CFPD quarters relative to CNT quarters. The strongest up-regulation among genes associated with TLR-signaling pathway (*CXCL8*, *interleukin 12A* (*IL-12A*) and *lipopolysaccharide binding protein* (*LBP*)), in CNT quarters was observed at 48 h post challenge. Both treatments showed significant down-regulation of *CXCL8* while *IL-12A* was only down-regulated in CF quarters and *LBP* was only significantly down-regulated in CFPD quarters, all in comparison to CNT quarters. Figure 2 summarizes treatment mediated down-regulation of selected genes grouped by their functional properties in the process of pathogen recognition signal transduction and cytokine and chemokine expression.

**Table 3:** Negative fold inductions of DE genes from challenged treated quarters compared to challenged not treated quarters associated to enriched pathways.

	24 h post challenge				48 h post challenge			
	Challenged cefapirin		Challenged cefapirin plus prednisolone		Challenged cefapirin		Challenged cefapirin plus prednisolone	
Gene Title	FI^a^	P-Value	FI^a^	P-Value	FI^a^	P-Value	FI^a^	P-Value
Chemokine (C-C motif) ligand 2 (CCL2)	–	–	−2.6	0.0015	–	–	–	–
Chemokine (C-C motif) ligand 3 (CCL3)	−4.3	0.0113	−4.7	0.0070	−2.5	0.0103	−2.5	0.0152
Chemokine (C-C motif) ligand 4 (CCL4)	−2.8	0.0063	−3.0	0.0039	–	–	–	–
Chemokine (C-C motif) ligand 5 (CCL5)	−2.3	0.0053	−2.1	0.0441	–	–	–	–
Chemokine (C-C motif) ligand 8 (CCL8)	–	–	–	–	−2.3	0.0108	−2.4	0.0126
Chemokine (C-C motif) receptor 1 (CCR1)	–	–	–	–	−2.4	0.0017	−2.4	0.0029
Chemokine (C-C motif) receptor 5 (CCR5)	–	–	–	–	−1.7	0.0265	−1.7	0.0315
CD molecule 40 (CD40)	–	–	–	–	−1.3	0.0116	−1.1	0.0315
Colony stimulating factor 1 receptor (CSF)	–	–	–	–	−1.4	0.0136	−1.1	0.0315
Chemokine (C-X-C motif) ligand 2 (CXCL2)	–	–	−1.0	0.0106	–	–	–	–
Chemokine (C-X-C motif) ligand 8 (CXCL8)	–	–	–	–	−3.0	0.0433	−3.3	0.0334
Chemokine (C-X-C motif) receptor 4 (CXCR4)	–	–	–	–	−1.7	0.0069	−1.7	0.0109
interleukin 12A (IL-12A)	−1.9	0.0012	–	–	−4.1	0.0344	–	–
lipopolysaccharide binding protein (LBP)	–	–	–	–	–	–	−2.8	0.0471
mitogen-activated protein kinase, kinase kinase 8 (MAP3K8)	–	–	–	–	−2.1	0.0103	−1.9	0.0243

^a^ log2 fold induction compared to challenged, not treated quarters.

### Validation of Selected Genes by Real Time qPCR

**Figure 3:**
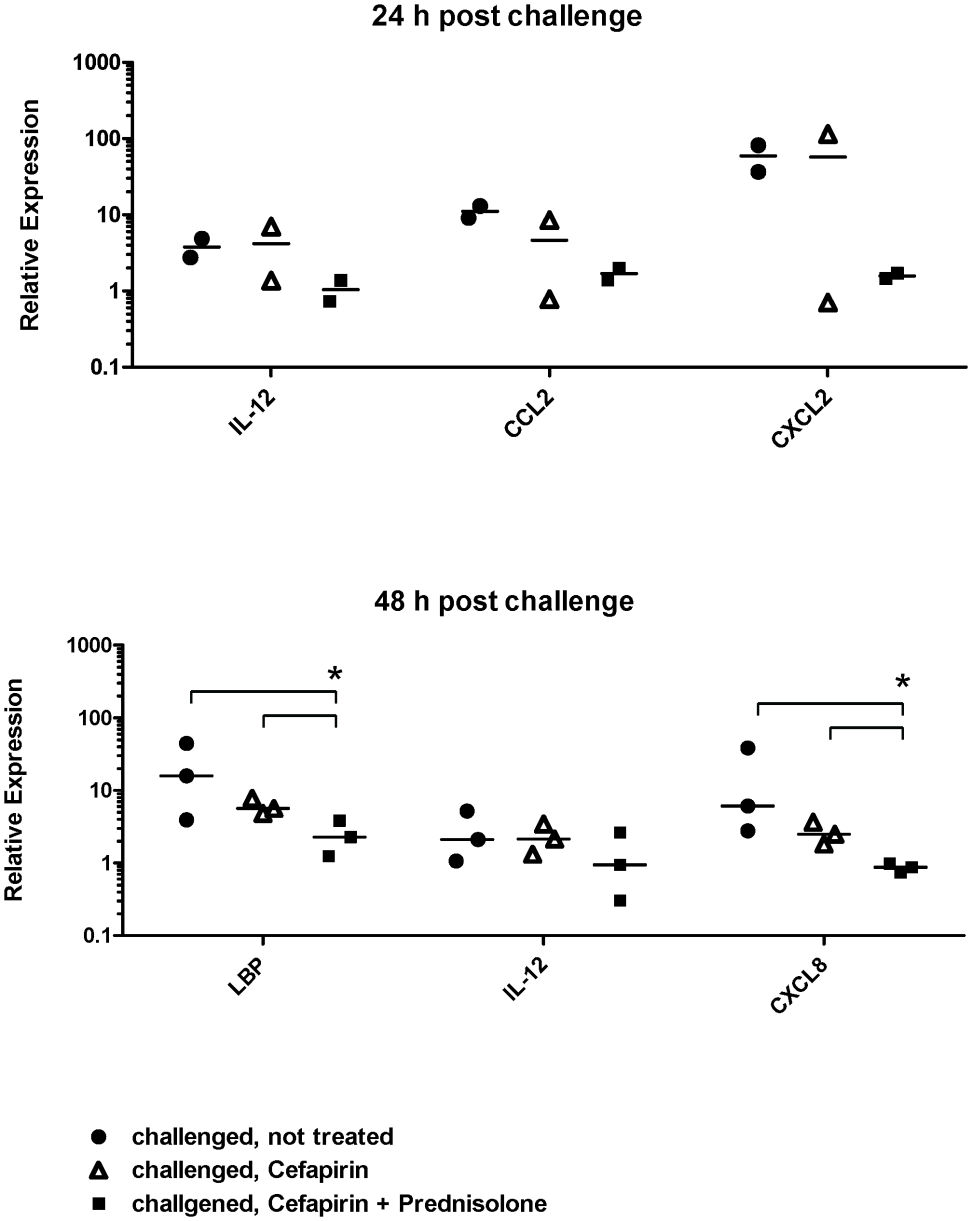
Validation of microarray results for selected genes by qPCR. Expression levels of DE genes in treated quarters belonging to significantly down-regulated pathways were confirmed by qPCR. Data are shown as median plus range of relative expression in challenged, not treated quarters (filled circle), challenged, cefapirin treated quarters (open triangle) and challenged, cefapirin plus prednisolone treated quarters (filled square). A) Samples obtained 24 h post challenge (n = 2). B) Samples obtained 48 h post challenge (n = 3). Statistically significant differences (P<0.05) are indicated by *.

The microarray results were validated by qPCR of the genes that were differently expressed in treated quarters at the respective time points representing the significantly regulated pathways. For the chemokine signaling pathway, *CCL2, CXCL2* and *CXCL8* were analyzed. For the TLR- signaling pathway, *LBP* and *IL-12A* were measured. Figure 3 shows relative expression values compared to NC quarters for the two sampling time points. Relative expression appeared to be lowest in CFPD treated quarters for all tested genes. For *CCL2* and *CXCL2* this is in line with the microarray result (Fig. 3 A). Interleukin-12A qPCR data at 24 h post challenge is in conflict with the microarray result (Fig. 3 A). However, none of the differences were statistically significant for the 24 h time point. At 48 h post challenge relative expression of *LBP* and *CXCL8* was significantly down-regulated in CFPD treated quarters compared to CNT quarters (P = 0.040) as well as compared to CF quarters (P = 0.0404) (Fig. 3 B). Expression of *IL-12A* analyzed by qPCR was not differentially regulated compared to CNT quarters at 48 h post challenge (Fig. 3 B). This result is in conflict to the microarray analysis where *IL-12A* expression was down-regulated in CF quarters.

### Chemokine Profiles in Milk Whey and Cell Influx Dynamics Confirm Microarray Results

**Figure 4:**
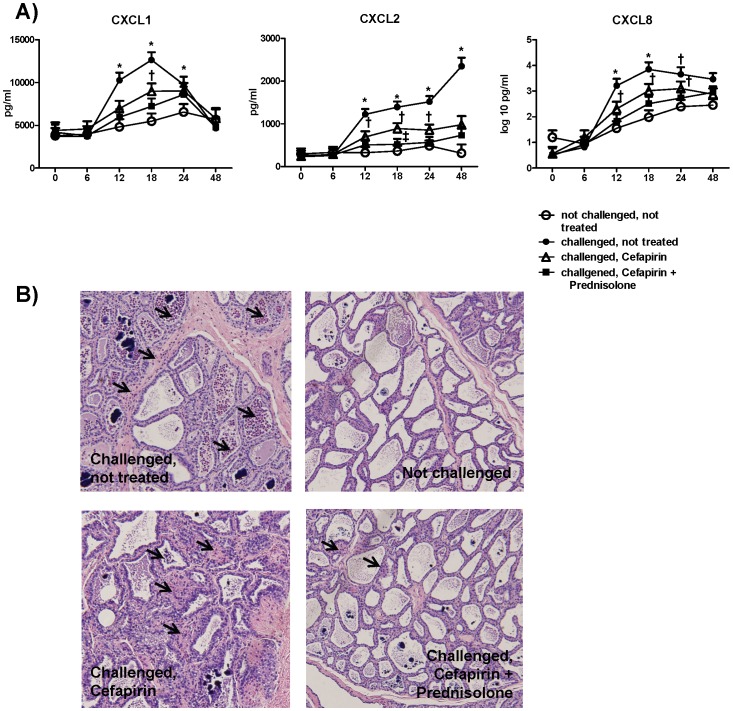
Levels of chemokines in milk whey. A) Levels (pg/ml) of chemokines (CXCL1, CXCL2 and CXCL8) in milk whey were determined by ELISA at time of (0), 6, 12, 18, 24 (n = 6) and 48 h (n = 3) post challenge with 100 cfu *E. coli*. Per cow one quarter was left unchallenged (open circles) and one quarter was challenged but not treated (filled circles), one quarter was challenged and cefapirin treated (open triangles) and one quarter was challenged and treated with cefapirin plus prednisolone (filled squares) at 4, 12 and 24 h post challenge. Data are shown as LSM of chemokine levels plus standard errors. In the case of CXCL8 LSM of the log10 transformed levels are shown. Statistically significant differences (P<0.05) are indicated by * (significantly different to all other groups), **†** (significantly different to not infected, not treated) and **‡** (significantly different to infected, cefapirin treated). B) Example of H&E stained sections from four quarters of one animal at 24 h post challenge. Arrows indicate PMN in tissue.

In CNT quarters all measured chemokines were significantly increased compared to NC as well as to challenged treated quarters. The observed increase started at 12 h post challenge and remained significantly increased until 24 h post challenge for CXCL1 and CXCL8, CXCL2 levels remained significantly increased until the end of the trial (Fig. 4 A). Cefapirin only treated quarters showed significantly increased levels compared to NC quarters, but significantly lower levels compared to CNT quarters, at 12 h post challenge for CXCL1 and at 12 h to 24 h post challenge for CXCL2 and CXCL8 (Fig. 4 A). At 18 h post-challenge, CFPD quarters displayed significantly lower levels of CXCL2 relative to CF quarters (Fig. 4 A). For the remaining time points, chemokine levels for CFPD quarters were numerically in-between the levels of cytokines in CF and NC quarters without being significantly different from either group. The lower level of chemokines in treated quarters relative to CNT quarters is also reflected in density of PMN in mammary gland tissue (Fig. 4 B). The exemplary tissue sections of 4 quarters from one animal at 24 h post challenge show a higher density of PMN in the CNT and in the CF quarter, only few PMN in the CFPD quarter and no PMN in the NC quarter.

## Discussion

In the present study we report for the first time the effect of intramammary treatment in experimental *E. coli* mastitis on gene expression profiles in udder tissue. The challenge model is based on within cow comparisons and thereby eliminating the high individual variation among cows in *E. coli* mastitis [32]. As a consequence only local responses in the respective quarter were captured and systemic reactions cannot be taken in account. Despite this, in comparison to other studies [17], [18], relatively low number of DE genes (231 at 24 h and 290 at 48 h post challenge) in CNT quarters compared to NC quarters, there was a clear effect of experimental infection (Fig. 1, Tab. 2). The response to challenge is in line with previously published microarray based studies on the local gene expression profiles after experimental *E. coli* challenge [17], [18], showing an up-regulation of genes associated with immune response and down-regulation of genes related to fat metabolism (Tab. 2). Treated quarters show high similarity to NC quarters as demonstrated by hierarchical clustering (Fig. 1) and the absence of DE genes between these groups. Significantly elevated chemokine levels in milk whey and a significantly increased density of PMN in udder tissue of CNT and CF quarters proof the presence of a pro-inflammatory response to challenge in these quarters (Fig. 4 A, B). Compared to CNT quarters the top most down-regulated signaling pathways in treated quarters for both time points are corresponding to the events of pathogen recognition, cytokine signaling and leukocyte recruitment (Fig. 2) which are decisive for the clinical outcome of an *E. coli* mastitis [33], [34]. Most of the differentially expressed genes appeared to be down-regulated in both treatment groups compared to CNT quarters with the same or similar fold induction suggesting that the observed treatment effects were CF mediated (Tab. 3). *Interleukin 12-A* was the only gene that was exclusively down-regulated in CF only treated quarters at both time points according to microarray data. The qPCR results however were not in line with these findings (Fig. 3). The reference genes used for qPCR were chosen because of their known stable expression in mammary gland tissue [29]. These genes are however not identical to the reference genes on the Affymetrix bovine gene chip, which may explain some deviations of results. Three genes were only differentially regulated in CFPD quarters (*CCL2* and *CXCL2* at 24 h, *LBP* at 48 h post challenge, Fig. 2, Tab. 3). Quantitative real time PCR confirmed the microarray results for *CCL2*, *CXCL2* and *LBP*, although differences were not statistically significant for *CCL2* and *CXCL2* due to the small sample number and, especially in the case of *CXCL2*, the high variation between replicates (Fig 3 A, B). Lipopolysaccharide binding protein is an acute phase protein that binds LPS and together with soluble CD14 facilitates the recognition of LPS through TLR4 and enhances the LPS-mediated priming effect of neutrophils [35], [36], [37]. While CXCL2 is a classical neutrophil chemoattractant, CCL2 which was mainly associated with monocyte recruitment and was just recently shown to induce accumulation of neutrophils in mice [38], [39]. Based on the gene expression profile, the addition of prednisolone in local treatment with cefapirin seems to further down-regulate the inflammatory response in terms of cell activation through LBP and the recruitment of leukocytes through CCL2 and CXCL2. The impact of treatment on the expression of classical neutrophil chemokines was also examined at protein level in milk whey (Fig. 4 A). Besides CXCL2 two other classical neutrophil attractants, CXCL8 and CXCL1, which share the receptor CXCR2 with CXCL2 were measured [40]. In contrast to CXCL1 and CXCL2 levels of CXCL8 continuously increased in NC quarters starting with 12 h post challenge. This could be interpreted as a systemic effect of challenge, which has also been reported by others [18], [20]. Despite this systemic effect NC quarters show significantly lower CXCL8 levels compared to CNT and CF quarters from 12 to 24 h post challenge (Fig. 4 A). Differences between the two treatments were only significant for CXCL2 at 18 h post challenge, where CF only treated quarters showed higher levels than CFPD quarters. The two treatments differed however in their chemokine expression compared to NC quarters. Chemokine levels in CF quarters were significantly higher than in NC quarters at 18 h post challenge for CXCL1 and from 12 to 24 h post challenge for CXCL2 and CXCL8, while CFPD quarters showed no significant difference to NC quarters at any time (Fig. 4 A). Although the measured protein expression with peak levels and strongest differences between the quarters at 12 to 24 h post challenge does not directly correspond to gene expression profiles at 24 and 48 h post challenge they convey the same message. All three chemokines showed highest levels in CNT quarters followed by CF quarters and lowest levels in CFPD quarters, indicating that treatment affects recruitment of PMN, which also became visible in mammary gland tissue sections (Fig. 4 A, B).

The recruitment and presence of activated neutrophils in the mammary gland is crucial for the host’s defense against pathogens [41] but also promotes tissue damage through the release of reactive oxygen species [42], [43]. In our previously published work we identified the reduced density of neutrophilic granulocytes in milk and tissue as key effect for both treatments. Treatment with cefapirin alone significantly reduced PMN density in tissue at 24 and 48 h post challenge, addition of prednisolone induced a significantly stronger reduction compared to quarters treated with cefapirin alone [22]. Whether the effect of cefapirin is completely due to its bactericidal activity or to a modulation of the immune response is not clear. Others have shown a suppression of LPS induced cytokine production by a third generation Cephalosporin, which could also apply for the first generation Cephalosporin cefapirin [44], [45]. Studies investigating effects of local treatment with prednisolone on the immune response in cattle are rare. Cannizzo et al. [46] recently published gene expression profiles in thymus of beef cattle after oral treatment with 30 mg prednisolone showing changes in expression of pro- and anti-inflammatory mediators. There is no overlap however between the DE genes in CFPD quarters from this study, which is very likely due to the fact that the beef cattle were not challenged with a pathogen. Heifers experimentally infected with *Mannheimia heamolytica* showed earlier resolution of infection when subjected to combination treatment with oxytetracyclin and isoflupredone compared to treatment with the antibiotic alone [47]. The corticosteroid also prevented pulmonary lesions that arise secondary to the host inflammatory response in *M. haemolytica* infections and are attributed to PMN influx in lung tissue [48]. In the present study the addition of prednisolone resulted in lower chemokine levels and lower density of PMN in mammary gland tissue compared to cefapirin treatment only (Fig. 4 A, B), which could be indicative for a lower level of tissue damage in quarters subjected to combination treatment.

This study showed for the first time the effect of intramammary treatment on local gene expression profiles in a within cow challenge model with *E. coli*. Due to the small number of replicates some numerical differences between the two treatments were not statistically significant. The presented findings demonstrate that both treatments significantly decrease gene and protein expression of chemokines in the mammary gland and thereby cell recruitment, especially recruitment of PMN, in the early phase of the inflammatory response. The addition of prednisolone showed an overall stronger effect compared to treatment with cefapirin alone. Together with an efficient bactericidal effect, a reduction of PMN density in the udder could decrease tissue damage and promote restoration of the gland. Based on the findings of this study treatment of *E. coli* mastitis appears to have beneficial effects compared to self cure. A consequence of the experimental challenge model however, is an infection intervention much earlier than we would see under field conditions. To facilitate within cow comparisons we challenged multiple quarters per cow which imposed the risk of systemic illness. The early treatment was a necessary compromise to keep the inflammation on a local level and allow for between quarter comparisons. As we discussed before, this artificial early treatment at 4 h after challenge will likely benefit the impact of treatment on bacteriological cure [22]. Further studies have to be conducted to evaluate effects of the local treatment with cefapirin in combination with or without prednisolone under natural challenge scenarios and after the onset of clinical signs.

## Supporting Information

Table S1 **List of DE genes in CNT compared to NC quarters 24 h post challenge.**
(XLSX)

Table S2 **List of DE genes in CNT compared to NC quarters 48 h post challenge.**
(XLSX)

Table S3 **List of DE genes in CF compared to CNT quarters 24 h post challenge.**
(XLSX)

Table S4 **List of DE genes in CF compared to CNT quarters 48 h post challenge.**
(XLSX)

Table S5 **List of DE genes in CFPD compared to CNT quarters 24 h post challenge.**
(XLSX)

Table S6 **List of DE genes in CFPD compared to CNT quarters 48 h post challenge.**
(XLSX)
